# Next‐generation sequencing through multi‐gene panel testing for the diagnosis of a Chinese patient with atypical Cockayne syndrome

**DOI:** 10.1002/mgg3.2254

**Published:** 2023-08-17

**Authors:** Xinyi Wang, Yue Li, Anqi Zhao, Yumeng Wang, Qiaoyu Cao, Chaolan Pan, Ming Li

**Affiliations:** ^1^ Department of Dermatology, Xinhua Hospital Shanghai Jiaotong University School of Medicine Shanghai China; ^2^ Department of Dermatology Children's Hospital of Fudan University, National Children's Medical Center Shanghai China; ^3^ Department of Dermatology Huashan Hospital of Fudan University Shanghai China

**Keywords:** Cockayne syndrome, ERCC8, microdeletion, photosensitivity, premature aging

## Abstract

**Background:**

Cockayne syndrome (CS, OMIM #133540, #216400) is a rare autosomal recessive disease involving multiple systems, typically characterized by microcephaly, premature aging, growth retardation, neurosensory abnormalities, and photosensitivity. The age of onset is related to the severity of the clinical phenotype, which may lead to fatal outcomes.

**Methods:**

We report a 3‐year‐old girl who presented with photosensitivity, gait abnormalities, stunting, and microcephaly and showed atypical clinical classification due to mild clinical manifestations at an early onset age.

**Results:**

Next‐generation sequencing reveals the frameshift mutation (c.394_398del, p.Leu132Asnfs*6) and a novel microdeletion of *ERCC8* (exon4del, p.Arg92fs).

**Conclusion:**

Therefore, it is still necessary to carry out next‐generation sequencing for CS patients with atypical clinical manifestations, which is essential for diagnosis and accurate genetic counseling.

## Graphical abstract

A novel microdeletion exon4del was found in a Chinese proband who displayed atypical CSA manifestations. More clinical and molecular data and molecular structure analysis are needed to further study their correlation.

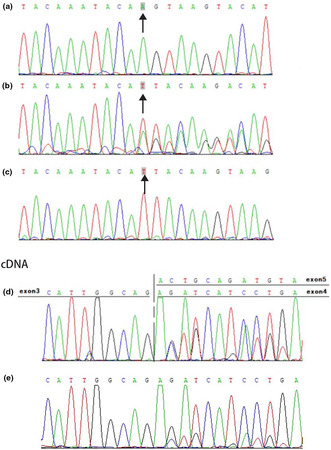

## 1 INTRODUCTION

Cockayne syndrome (CS, OMIM #133540, #216400) is a rare autosomal recessive disease involving multiple systems, which was first reported by Edward Cockayne in 1936 (Cockayne, 1936; Karikkineth et al., 2017). Typical clinical manifestations include growth retardation, dwarfism, underweight, photosensitivity, tooth decay, microcephaly, special facial features, premature aging, and subcutaneous fat loss. Patients show increasing lower limb tension and abnormal gait due to hyperreflexia and limited ankle dorsiflexion. Neurological examinations reveal spastic paraplegia and cerebellar ataxia (Hashimoto et al., 2008; Kubota et al., 2015; Laugel, 2013; Wilson et al., 2016). Accelerated hypertension, renal failure, and abnormal gastrointestinal motility are also of possibility (Ovaert et al., 2007; Wilson et al., 2016). CS is divided into three types (Nance & Berry, 1992). CSI is typical and usually has an onset in early childhood. CSII occurs at birth and presents with severe growth defects and neurodevelopmental disorders, and patients usually die at the age of 6 or 7. CSIII usually starts in late childhood, with manifestations gradually appearing with age.

Previous studies reveal that CS is caused by homozygous or compound heterozygous mutations in two genes, *ERCC6* or *ERCC8* (Laugel et al., 2010). ERCC encodes the protein transcription‐coupled nucleotide excision repair (TC‐NER) involved in UV‐induced DNA damage (Venema et al., 1990).

Because of the poor prognosis and high mortality risk of CS, early diagnosis of the disease is particularly important. We report a novel microdeletion mutation and a recurrent frameshift mutation in the *ERCCR* gene in a 3‐year‐old girl with atypical CS manifestations.

## 2 CASE REPORT

**FIGURE 1:**
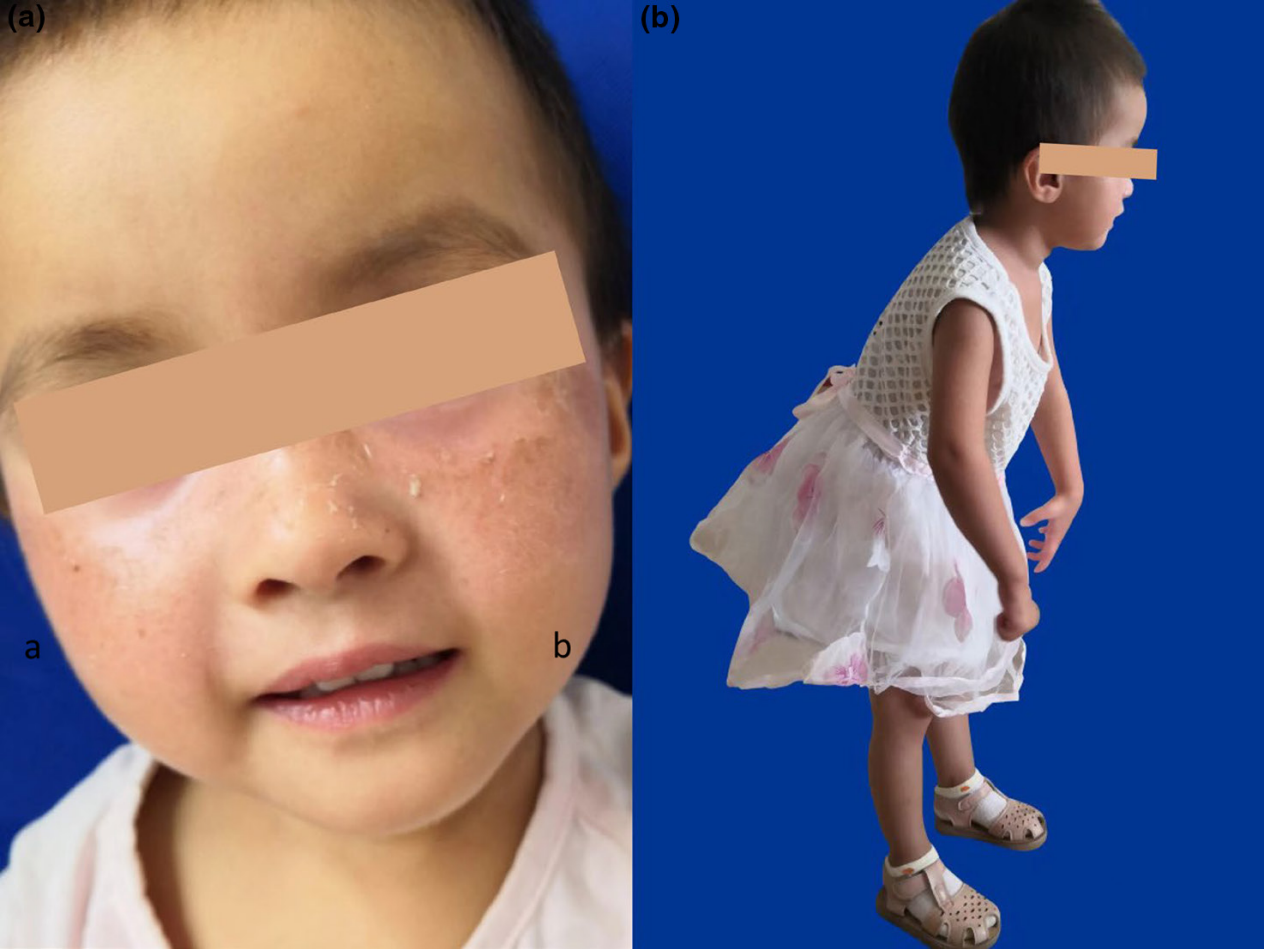
Manifestations of the patient. (a) Erythema, scaling, and black nevus on both cheeks and other light‐exposed areas. (b) The patient displayed a scissors gait.

**FIGURE 2:**
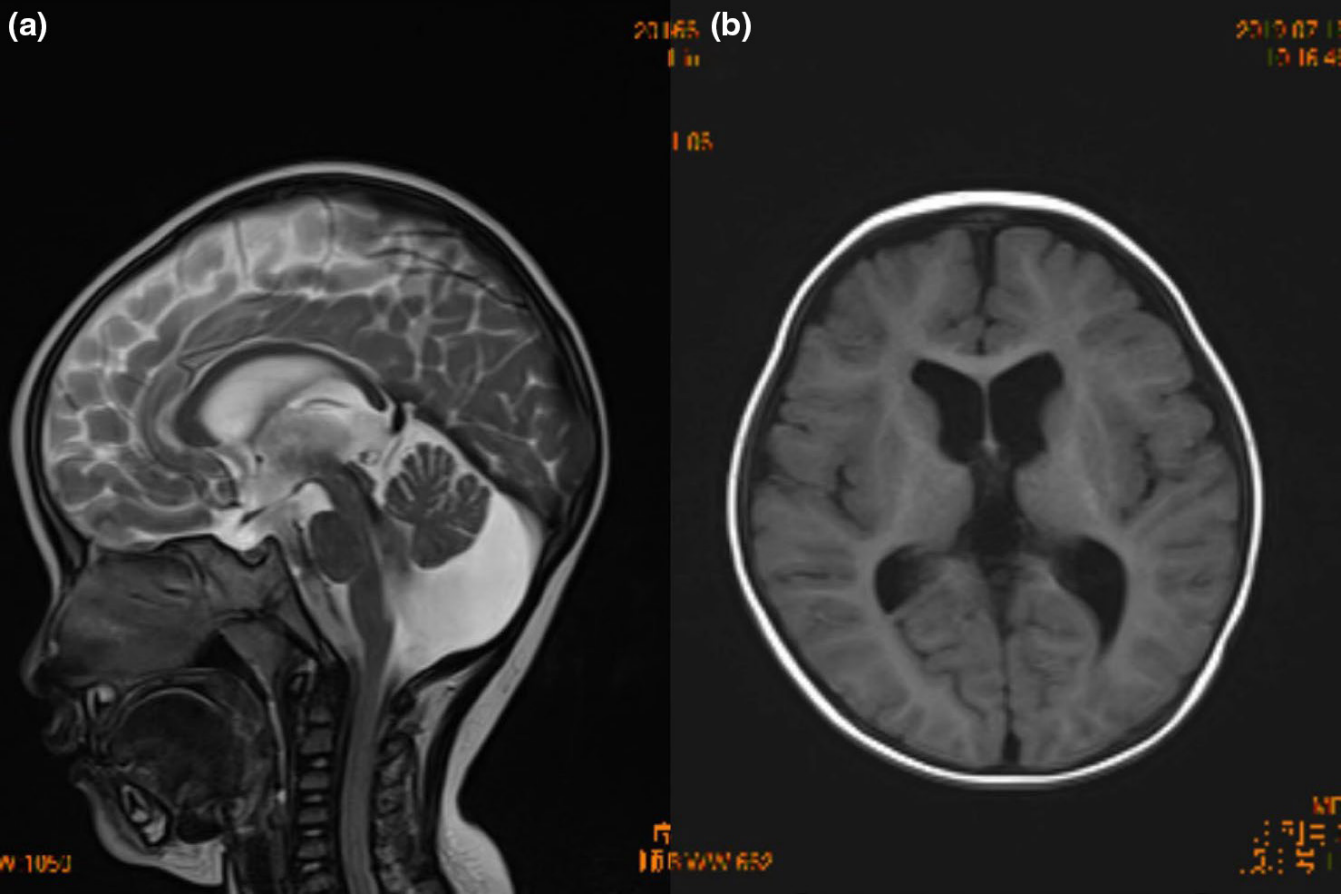
The brain magnetic resonance imaging (MRI) of the patient. The bilateral ventricles and the third ventricle were widened, (a) the midbrain aqueduct was not clear, (b) the lateral fissure cistern on both sides was slightly widened, the left temporal base extracerebral space was widened, and mega cisterna magna was found.

A 3‐year‐old girl came to the doctor, suffering from sustained erythema, scaling, and other light‐exposed parts shortly after birth (Figure 1a). She showed poor gastric appetite, low myodynamia, scissors gait, and low crying voice (Figure 1b). Her weight was 10.3 kg (P3), with a height of 88.7 cm (P15), and a head circumference of 45.7 cm (P3), indicating obvious underweight and microcephaly compared with others of the same age. According to the Gesell intelligence test, the DQ scores of gross motor function, physical function, and human function were 45 (equivalent to 14.5 months of age), that of fine motor function was 46 (equivalent to 15 months of age), speech function's was 39 (equivalent to 12.5 months of age), and an edge of social adaptability suggested that the children had mental retardation. The brain magnetic resonance imaging of the patient showed several ventricles widened (Figure 2). No obvious abnormality was found in EEG and BEAM. X‐ray showed that the continuity of left Shenton's line was not good, indicating subluxation or internal rotation deformity of the hip joint, consistent with her standing posture.

When she turned 6 years old, she could enunciate phrases composed of four syllables. Erythema has disappeared over a long period of being at home. She has six deciduous teeth missing for 3 years.

The parents of the proband are not consanguineous, and her four sisters are normal. Her mother miscarried a boy during pregnancy without prenatal screening.

## 3 MATERIALS AND METHODS

According to the requirements of the Ethics Committee of Xinhua Hospital affiliated with the Medical College of Shanghai Jiao Tong University, the patient's family members gave informed consent before receiving DNA testing, and the test was carried out according to the Helsinki Declaration.

Genomic DNA was extracted from the collected blood samples of the proband, and randomly broken into fragments with a length of 150–250 bp by Covaris ultrasonic crusher. After the end repair and adding A tail, the two ends of the fragments were connected with Y joints to prepare a DNA library. After pooling the DNA library with different index tags, the probe with biotin tags is hybridized in the liquid phase, and then all exons of the gene to be tested are captured with a magnetic bead coated with streptavidin. After linear amplification by PCR, the library quality is checked, and the qualified library can be sequenced with high throughput.

The probe used covers more than 500 genes closely related to hereditary dermatosis. IlluminaHiseq X Ten is selected as the sequencing platform, with an average sequencing depth of more than 200X and Q30 > 90%.

Illumina CASAVA1.8 is used to convert bcl files into fastq files for the original data of sequencer offline, and BWA, Samtools, and Picard software are used to compare Reads to the human reference genome GRCh37/hg19. The generated bam file was partially re‐aligned with GATK series software, and the mutation was detected after the duplicate sequence was removed. Use Anovar to annotate the VCF variation files, and then screen them according to their frequency, location, functional consequences, genetic model, and more important clinical phenotype.

Sanger sequencing was carried out for verification of the proband's parents. Four separate reaction systems were established, including four deoxynucleotide triphosphates, DNA polymerase, four isotope‐labeled dideoxynucleoside triphosphates, target fragments, and sequencing primers. The target fragment was used as a template, and DNA polymerase was used as a catalyst to start DNA replication from the primer. The reaction stopped when it encountered dideoxynucleoside triphosphate. Then we prepared 1.5% agar gel, separated the above reaction products by gel electrophoresis, and used X‐ray gel autoradiography for inspection. The longitudinal regular bands were recognized as electrical signals by the computer through light excitation, and the data were read with the help of DNA sequencing analysis technology.

Geneious was used to translate original and mutated base sequences into amino acid sequences. Then we used the monomer_casp14 model in Alphafold v2.3.1 with default parameters to predict the structures of mutated proteins, using the following databases: uniport, uniref90 v.2023‐03‐01, pdb_mmcif, pdb_Seqres v.2023‐03‐03, and other default versions.

## 4 RESULTS

**FIGURE 3:**
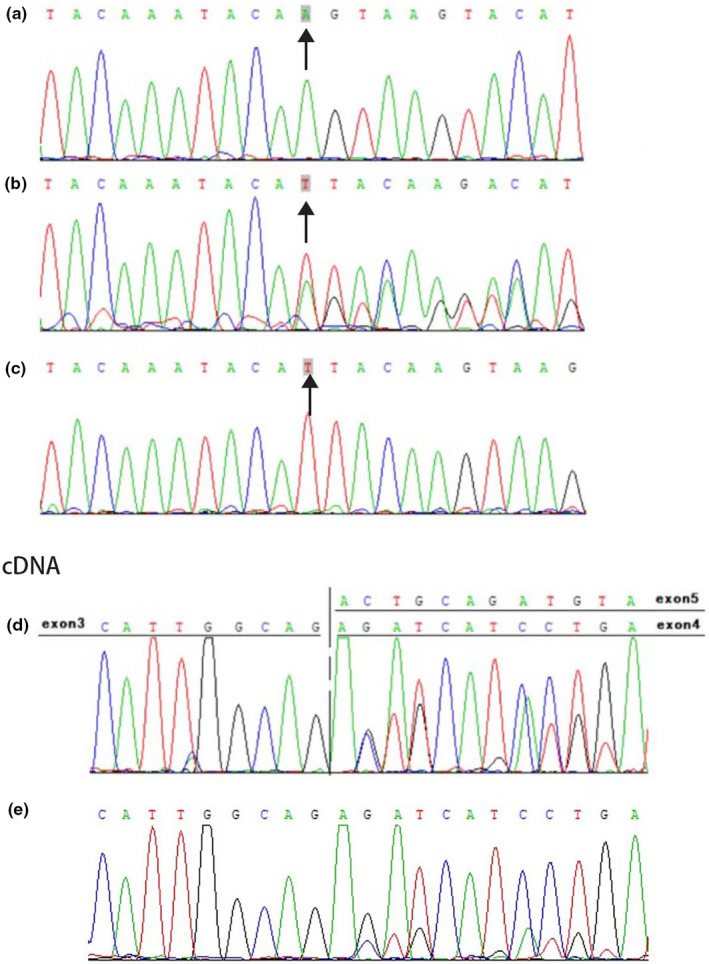
DNA verification and cDNA verification. (a) The proband carries the compound heterozygous mutation of c.394_398del. Due to the deletion of alleles, the sequencing results showed as homozygous. (b)The proband's father carries c 394_398del. (c)The mutation c 394_398del was not found in the blood sample of the proband's mother. (d, e) The cDNA verification showed that both the proband and her mother carried the microdeletion of exon4del on mRNA.

Results showed compound heterozygous variations of c.394_398del (p.Leu132Asnfs*6) and exon4del (p.Arg92fs) in *ERCC8* (Figure 3). Parental verification showed that two variations of the subject were, respectively, from the father and mother of the proband.

**FIGURE 4:**
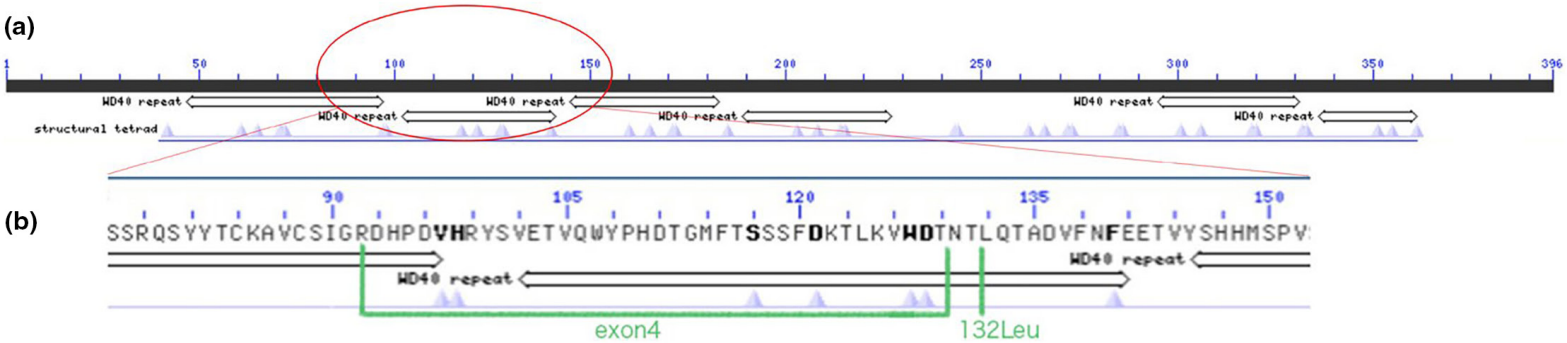
(a) The normal protein sequence is composed of several conserved domains.(b) Zoom to residue level, the variant c.394_398del (p.Leu132Asnfs*6) and most of exon4del (p.Arg92fs) are within the conserved domain WD40 repeat.

The variant c.394_398del(p.Leu132Asnfs*6) is in the conserved domain, and most of the proteins translated by exon4del (p.Arg92fs) are within the conserved domain (Figure 4), which indicates both the two variants are pathogenic.

**FIGURE 5:**
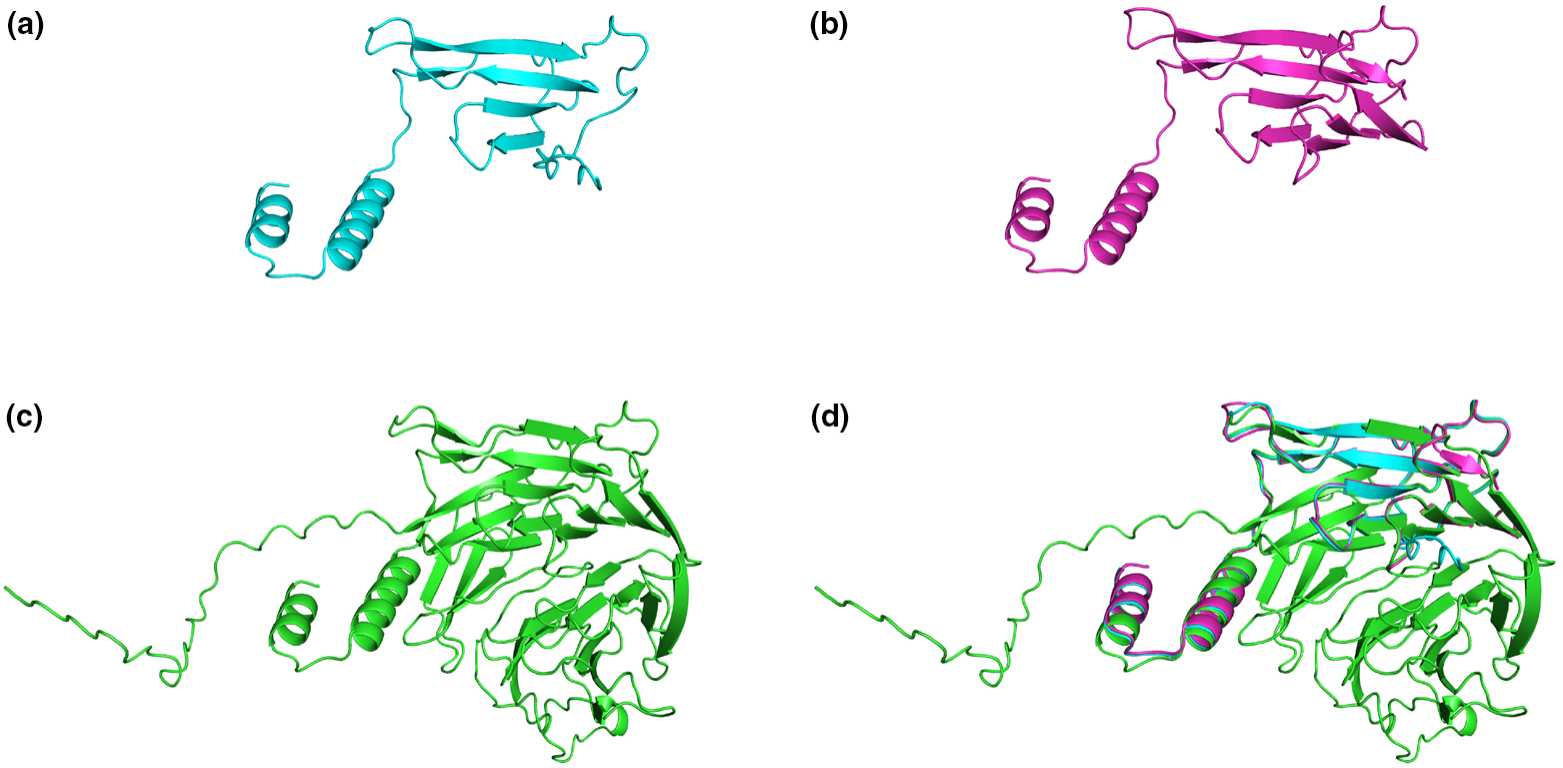
Protein structures before and after mutations. (a) The predicted structure of exon4del (p.Arg92fs) is composed of 117 amino acids and then stops, with the same sequence in the first 106 amino acid sequences compared with the original one. The two structures are very similar (RMSD = 0.452). (b) The predicted structure of c.394_398del (p.Leu132Asnfs*6) is composed of 136 amino acids. (c) The original sequence is composed of 396 amino acids. (d) Both mutations result in protein truncation, and they are similar in structure to the original sequence in the first 106 amino acid sequences (RMSD = 0.452) and 131 amino acid positions (RMSD = 0.339), respectively. The two structural differences did not result in significant differences before and after the mutation, nor did they affect the local structure of the protein.

Both mutations result in protein truncation (Figure 5). NM_000082.4:c.394_398del (p.Leu132Asnfs*6) is a frameshift mutation, which would cause the coding protein to terminate early in exon 4. This variant leads to the mutation of amino acid 132, and the subsequent amino acid sequence probably shift to produce a truncated protein. At the mRNA level, nonsense‐mediated mRNA decay (NMD) may block the expression of truncated proteins with potential toxicity by recognizing and degrading transcripts containing the premature translational‐termination codon (PTC). This process can relieve or alleviate the phenotype of diseases in most cases. However, when truncated protein residues caused by PTC have some normal physiological functions, the effect of NMD would aggravate the disease phenotype.

The other variant is a gross deletion of the genomic region encompassing exon(s) 4 of the ERCC8 gene. This deletion is out‐of‐frame and is expected to create a premature termination codon and result in an absent or disrupted protein product. Both c.394_398del(p.Leu132Asnfs*6) and exon4del (p.Arg92fs) exhibit irregular curls that differ from the original sequence after amino acids 132 and 108, respectively. Subsequent protein structures including α‐helix, β sheet, and the loop are speculated lost, resulting in a large loss of the three‐dimensional structure of the protein, which would lead to function loss of the encoded protein.

## 5 DISCUSSION

In CS, NER of oxidized genomic DNA, which eliminates DNA damage caused by ultraviolet or chemical radiation and maintains normal replication or transcription, leads to premature aging (Venema et al., 1990). Several other roles have been found over the years, making CS not only a DNA repair disorder but also a severe transcription initiation defect (Proietti‐De‐Santis et al., 2006). Indeed, they participate in basal and activated transcription and in the recovery of RNA synthesis after the massive transcriptional shutdown induced by genotoxic stresses (Epanchintsev et al., 2017; Kristensen et al., 2013), as well as in the ubiquitination/degradation of key targets such as p53 (Proietti‐De‐Santis et al., 2006), thus re‐equilibrating the physiological response in favor of cell survival and proliferation instead of cell cycle arrest and cell death, and PRC1 (Paccosi et al., 2020), thus regulating cell cycle progression. Moreover, they are involved in mitochondrial homeostasis (Chatre et al., 2015; Scheibye‐Knudsen et al., 2012) and in the mediating transcription of RNA polymerase I for ribosomal biogenesis (Okur et al., 2020). At least 20–30 proteins are considered to play a key role in this pathway in a sequential manner. The DNA repair disorder of CS is caused by *ERCC6* or *ERCC8* with a proportion around 1:2 (Kou et al., 2018). *ERCC8* is located in 5q12.1, coding the WD repeat protein where all the reported significant missense mutations are located (Calmels et al., 2018), which indicates that the integrity of the WD domain plays a key role in the biological function of CSA.

Up to now, at least 91 mutations have been found in the *ERCC8* gene, composed of missense mutations (35.2%), nonsense mutations (15.4%), intron mutations (5.5%), splicing mutations (15.4%), frameshift mutations (14.3%), and synonymous mutations (14.2%), showing the distribution of different types of *ERCC8* gene mutations. These mutations are distributed along the entire genome sequence and represent most types of mutations. Clinical manifestations may be related to the genotype. Children with nonsense mutation and frameshift mutation may be of more typical symptoms, while those with missense mutation are of less symptoms. The mutation at 5′ end causes fewer symptoms than that at 3’ end. These results suggest the heterogeneity of CS phenotype.

In this case, the proband's father brought her a frameshift mutation (c.394_398del, p.Leu132Asnfs*6), but as a carrier, he did not show any clinical phenotype. This mutation is a sub‐high frequency mutation in Chinese patients with CS‐A (Wang et al., 2017). Clinical phenotype caused by truncated or abnormal CSB protein is more serious than that caused by complete deletion of CSB protein, and homozygous mutations may lead to a more serious phenotype (Proietti‐De‐Santis et al., 2006). However, no similar situation has been found in CS‐A research. The other mutation is a partial deletion of the *ERCC8* gene caused by 5q12.1 microdeletion, named exon4del(p.Arg92fs), from the mother of the proband. Subchromosomal deletions with a size of less than 50 kb are usually called microdelections (sub microscopical losses). When unbalanced regions appear in key genes or important regulatory regions, microdeletions tend to produce phenotypic effects (Levy & Wapner, 2018). In this case, the mutation gives rise to the loss of exon 4 composed of 124 bases on mRNA, producing a truncated protein, thus causing disease. A study shows that the exon 4 rearrangement mutation is a founder mutation of East Asian people (Wang et al., 2017).

Considering that the proband's EEG and ophthalmic examination were not significantly abnormal, and the short stature was not obvious, we assume that the manifestations are not typical. and the patient can not be simply divided. In addition, the patient was born with onset, which was consistent with CSII, but the severity of the phenotype was more likely to be classified as CSI, revealing an atypical manifestation of CS. Studies have found that in homozygous patients with CS‐A, missense mutations frequently lead to mild phenotypes than changes in protein truncation (Calmels et al., 2018). In addition to pathogenic mutations, additional disease modifiers can influence penetrance. This may explain the occurrence of incomplete penetrance. In order to study the significance of their determination, more experiments are needed.

A clear diagnosis of CS is essential to provide accurate prognosis guidance, genetic counseling, and prenatal diagnosis (Duong et al., 2022). Although heterozygous carriers do not have obvious clinical manifestations of the disease, they are at risk of passing this harmful mutation on to their offspring. Improving the mutation spectrum of the disease is conducive to a better understanding of CS.

In conclusion, a novel microdeletion exon4del was found in a proband who displayed atypical CSA manifestations. More clinical and molecular data and molecular structure analysis are needed to further study their correlation.

## AUTHOR CONTRIBUTIONS

Xinyi Wang and Ming Li were responsible for the conceptualization of the study. Yue Li coordinated patient recruitment, clinical data collection, and sample collection. Anqi Zhao, Yumeng Wang, Qiaoyu Cao, and Chaolan Pan performed the sequencing and analysis of the results. Xinyi Wang wrote the initial draft. All authors contributed to reviewing and editing the manuscript. Yue Li supervised the analyses. Ming Li supervised the draft of the manuscript.

## CONFLICT OF INTEREST STATEMENT

None declared.

## ETHICS STATEMENT

According to the requirements of the Ethics Committee of Xinhua Hospital affiliated to the Medical College of Shanghai Jiao Tong University, the patient's family members gave informed consent before receiving DNA testing, and the test was carried out according to the Helsinki Declaration.

## Data Availability

The data that support the findings of this study are available from the corresponding author upon reasonable request.
